# Supplementary material for: Environmental monitoring and health assessment in an industrial town in central India: A cross-sectional study protocol
Source: PLoS One. 2022 Jun 16;17(6):e0264154. doi: 10.1371/journal.pone.0264154 (PMC9202949; doi:10.1371/journal.pone.0264154)
Supplement: Attachment — Submitted filename: PONE-D-22-03418.pdf [file pone.0264154.s001.pdf]

Title: Environmental monitoring and health assessment in an industrial town in central India: A cross-sectional study protocol

Short Title: Environmental public health assessment in an Indian industrial town

Manuscript No.: PONE-D-22-03418

Authors: Tanwi Trushna, Vikas Dhiman, Satish Bhagwatrao Aher, Dharma Raj, Rajesh Ahirwar, Swasti Shubham, Subroto Shambhu Nandi, Rajnarayan R Tiwari

General comments

The submitted manuscript is a protocol of a proposed environmental health study involving multiple parameters. Though the traditional standard methods for analysis/assessment of various individual parameters are already known, the comprehensiveness and wide scope of the study is a successful novel attempt. The huge number of study participants, clinical examinations, biological assessment (blood and urine), sampling and analysis designed for environmental matrices (air and water), lung function test, ECG, neurological assessment proposed by the authors is a piece of wonderful work and will serve as a ready reckoner for future environmental health studies.

Though the proposed protocol is for conducting environmental health study in and around man-made fibre and allied industrial settlement, the same can be used for diverse sectors with little modifications considering the type and intensity of environmental contaminants.

Specific comments:

Line No. 207: Monitoring of metallic pollutants in groundwater:

The sample processing/digestion techniques and equipment/instrument proposed to use is missing, include a brief description.

Line No. 226-227: Provide the Reference for Detection of chloride and sulphate will be carried out using titration methods.

Line No. 234-233: Mention the approximate duration of storage.

Line No. 239: m ???? Do you mean mL ?

Line No. 240: Provide the reference.

Line No. 412: Of. Change to of

Line No. 416: In 1st. It should be in 1st

Line No. 417: Hcl. It should be HCl

Line No. 421: Kg/m2 – Kilogram Per Square Metre. No where found in entire manuscript. Delete

Line No. 435 & 436: SA or SBA?? Maintain the uniformity

1. Use uniform styles of formatting in abbreviation.
2. Strictly follow the author guidelines of the journal.

Recommendation: The manuscript is <u>recommended</u> for publication after minor modifications.
